# Pseudoaddiction: Fact or Fiction? An Investigation of the Medical Literature

**DOI:** 10.1007/s40429-015-0074-7

**Published:** 2015-10-01

**Authors:** Marion S. Greene, R. Andrew Chambers

**Affiliations:** Center for Health Policy, Richard M. Fairbanks School of Public Health, Indiana University-Purdue University Indianapolis (IUPUI), 714 N Senate Ave, Indianapolis, IN 46202 USA; Laboratory for Translational Neuroscience of Dual Diagnosis & Development, Department of Psychiatry, IU Neuroscience Center, Indiana University School of Medicine, 320 W. 15th Street, Indianapolis, IN 46202 USA

**Keywords:** Pseudoaddiction, Iatrogenic, Opioid addiction, Chronic pain, Prescription drug epidemic

## Abstract

Tremendous growth in opioid prescribing over two decades in the USA has correlated with proportional increases in diversion, addiction, and overdose deaths. Pseudoaddiction, a concept coined in 1989, has frequently been cited to indicate that under-treatment of pain, rather than addiction, is the more pressing and authentic clinical problem in opioid-seeking patients. This investigative review searched Medline articles containing the term “pseudoaddiction” to determine its footprint in the literature with a focus on how it has been characterized and empirically validated. By 2014, pseudoaddiction was discussed in 224 articles. Only 18 of these articles contributed to or questioned pseudoaddiction from an anecdotal or theoretical standpoint, and none empirically tested or confirmed its existence. Twelve of these articles, including all four that acknowledged pharmaceutical funding, were proponents of pseudoaddiction. These papers described pseudoaddiction as an iatrogenic disease resulting from withholding opioids for pain that can be diagnosed, prevented, and treated with more aggressive opioid treatment. In contrast, six articles, none with pharmaceutical support, questioned pseudoaddiction as a clinical construct. Empirical evidence supporting pseudoaddiction as a diagnosis distinct from addiction has not emerged. Nevertheless, the term has been accepted and proliferated in the literature as a justification for opioid therapy for non-terminal pain in patients who may appear to be addicted but should not, from the perspective of pseudoaddiction, be diagnosed with addiction. Future studies should examine whether acceptance of pseudoaddiction has complicated accurate pain assessment and treatment, and whether it has contributed to or reflected medical-cultural shifts that produced the iatrogenic opioid addiction epidemic.

## Introduction

Over the last 20 years, US health care has undergone a major shift in attitudes and clinical practice involving increased prescribing of opioids to patients with different causes and levels of acute and chronic pain. This shift has been attributed to various trends and organizational initiatives within health care that have (1) increased the prioritization and emphasis on the diagnosis of pain, relative to other medical issues (e.g., “pain is the fifth vital sign”) [1], and (2) the treatment of pain with opioid analgesics as opposed to other modalities that have reputations of being less clinically efficacious and/or less cost-effective than opioids [2•]. Accordingly, massive increases in US expenditures for prescription pharmaceuticals (from $12 billion in 1980 to $263 billion in 2011 [3]) have been driven in part by increases in opioid prescriptions and consumption [4–9]. From 2000 to 2009, opioids dispensed from US outpatient pharmacies rose from 174.1 million to 256.9 million doses [10]. By the end of the last decade, Americans, although just 4 % of the world’s population, were consuming 80 % of the global opioid supply and 99 % of the world’s hydrocodone supply [9, 11].

While the origins of these trends are attributable to the recognition of pain as a significant, undertreated public health problem [12–15], a growing emphasis on opioids as the major, front-line treatment of choice for pain has gone hand in hand with reducing physicians’ fears of opioids causing or contributing to addiction [16, 17]. Pseudoaddiction, the subject of this review, is a clinical concept that has been influential as a diagnosis in clinical practice and the medical literature to indicate that under-treatment of pain, rather than risk of addiction with opioids, should be the primary clinical concern. Pseudoaddiction (with “pseudo-” from Latin meaning “fake,” “not real,” www.Merriam-Webster.com) was originally introduced and defined by Weissman and Haddox in 1989 as an “iatrogenic syndrome that mimics the behavioral symptoms of addiction” in patients receiving inadequate doses of opioids for pain [18].

Unfortunately, in the quarter century after the introduction of pseudoaddiction, sharp rises in the utilization and supply of opioids via prescribers in the USA have been paralleled by rises in negative outcomes, including abuse and diversion, emergency department encounters, addictions, and fatal overdoses [6, 8, 9, 19•, 20]. For example, as emergency department visits related to opioid overdoses increased from 144,655 in 2004, to 366,181 in 2011 [21], their fatal outcomes quadrupled from 4030 in 1999, to 16,651 in 2010 [22••]. In 2010, not accounting for any treatment for opioid addiction, US hospital costs for treating opioid overdoses was four times larger than for heroin overdoses and totaled nearly $2.3 billion—roughly double the annual extramural research budget of the National Institute on Drug Abuse [23••]. In line with this data, the Centers for Disease Control and Prevention (CDC) and the Office of National Drug Control Policy (ONDCP) have listed prescription drug overdoses as an epidemic [24].

The purpose of this review is to describe the scope and content of the medical literature on pseudoaddiction since its introduction a quarter century ago. The origins of pseudoaddiction as a diagnostic construct, its sponsorship via pharmaceutical industry support, its empirical basis, and its proponent and opponent arguments are covered. We then conclude with a discussion of implications of these findings in terms of the current prescription opioid epidemic, with considerations for future research. This examination is relevant and timely to the modern prescription opioid epidemic, given the fact that the vast majority of opioid medications that are misused and/or diverted to generate addiction and overdoses do originate as legal prescriptions from doctor’s offices [25, 26]. Prolific over-prescribing of opioids has grown so routine that, in at least one US state, geographical proximity to medical care and outpatient pharmacies that dispense opioids has been demonstrated to be a population risk factor for acquiring addictive disease [19•]. As contextualized by health insurance reimbursement and medication coverage plans that readily support opioid prescribing for pain diagnoses, while remaining relatively unsupportive of evidence-based treatments for addiction diagnoses [2•, 27•], these trends suggest that since the introduction of pseudoaddiction, health care has shifted too far in favor of treating pain with opioids. With a new and broadening consensus that the evidence base does not support the use of chronic opioids for chronic, non-terminal pain [4, 5, 15, 17, 28–32, 33••], a literature review of pseudoaddiction is needed to inform opinions on whether the construct has been harmful or beneficial to patients, and whether it should survive or be abandoned.

## Origination of Pseudoaddiction

The 1989 introduction of pseudoaddiction happened in the form a single case report of a 17-year-old man with acute leukemia, who was hospitalized with pneumonia and chest wall pain [18]. The patient was initially given 5 mg of intravenous morphine every 4 to 6 h on an as-needed dosing schedule but received additional doses and analgesics over time. After a few days, the patient started engaging in behaviors that are frequently associated with opioid addiction, such as requesting medication prior to scheduled dosing, requesting specific opioids, and engaging in pain behaviors (e.g., moaning, crying, grimacing, and complaining about various aches and pains) to elicit drug delivery. The authors argued that this was not idiopathic opioid addiction but pseudoaddiction, which resulted from medical under-treatment (insufficient opioid dosing, utilization of opioids with inadequate potency, excessive dosing intervals) of the patient’s pain. In describing pseudoaddiction as an “iatrogenic” syndrome, Weissman and Haddox inverted the traditional usage of iatrogenic as harm caused by a medical intervention. In pseudoaddiction, iatrogenic harm was described as being caused by *withholding* treatment (opioids), not by providing it.

Weissman and Haddox proposed that patients who present with pseudoaddiction go through three phases: stimulus, escalation, and crisis. In stimulus, at pain onset, the patient receives inadequate analgesia and requests more medication, frequently requesting specific drugs by name. In escalation, the patient realizes that to receive additional medication, he has to convince a health care provider of the legitimacy of his or her pain. In crisis, culminating when unrelieved pain continues, the patient engages in increasingly bizarre drug-seeking behaviors, leading to “a crisis of mistrust” with anger and isolation by the patient and frustration and avoidance by the health care team. The authors concluded that to prevent or treat pseudoaddiction, the health care team needs to (a) establish trust, i.e., “the patient must have trust that the caregivers believe the pain is real and that all attempts will be made towards pain control,” and (b) provide rational pain management, i.e., appropriate and timely use of opioids based on patients’ pain reports.

## Footprint in the Medical Literature and Pharmaceutical Industry Support

A comprehensive search of the National Library of Medicine’s bibliographic database/article index (OVID Medline), to gauge the scope and content of the literature on pseudoaddiction, was conducted by the authors in November, 2013, entering “pseudoaddiction” as the key word. A total of 224 papers were identified. From this collection, 134 were review articles (including clinical reviews and guidelines, ethical and policy reviews, and position and consensus statements based on reviews), 33 were original studies (i.e., studies that collected and analyzed primary data), 31 were commentaries (including editorials, clinical notes, and chapter introductions), 10 were case reports, and 16 were categorized as “other” (including reference books, book reviews, abstracts, posters, and policy statements). Most of the entries (106) appeared in pharmacological, pain, anesthesiology, palliative care, and oncology journals. The rest were published in journals encompassing general medicine and related subspecialties (54), nursing (27), addiction and psychiatric journals (19), psychological/neurological journals (8), and other types of journals or books, including ethics and policy, health services research, nutrition, science, and reference books (10).

Further examination of this set of 224 papers classified them according to whether they were (1) studies that attempted to empirically validate pseudoaddiction as a clinical concept; (2) articles that examined the concept critically and added new thought to supporting, characterizing, or refuting the phenomenon; and (3) papers that accepted pseudoaddiction as a true clinical phenomena or diagnosis by restating previous definitions or providing explanations or examples without adding new knew knowledge or perspectives on it. Of the 224 articles, none exist that attempted to empirically validate the concept of pseudoaddiction (category 1). Just 18 provided supportive elaboration on the concept and/or added critical thought (category 2) while 206 papers cite the concept as a matter of routine acceptance. Among the 18 category 2 articles, 12 elaborated on pseudoaddiction as a genuine clinical phenomenon, 4 were in disagreement, and 2 addressed pseudoaddiction not from a clinical but a social perspective.

Because pharmaceutical company funding of academic medical publications or authors has been found to be associated with conclusions favoring those companies’ interests [34], we also determined if papers or their authors were noted as receiving pharmaceutical company support. Of the 224 publications, 22 were marked as being sponsored by pharmaceutical companies or having authors that received pharmaceutical honoraria. Pharmaceutical companies that provided this support and representative opioid products sold by them included the following: Abbott Laboratories (meperidine, various hydrocodone formulations, hydromorphone), Alpharma (morphine formulations), ALZA Corporation (fentanyl), Bristol-Meyers Squibb (oxymorphone formulations), Cephalon, Inc. (fentanyl formulations), Grünenthal GmbH (Tramadol), Janssen-Cilag/Johnson & Johnson Pharmaceuticals (fentanyl, tapendadol), King Pharmaceuticals (morphine formulations), Purdue Pharma (oxycodone, hydromorphone), and QRx Pty, Ltd. (morphine and oxycodone formulations). While the original 1989 Weisman and Haddox paper introducing pseudoaddiction did not list pharmaceutical support [18], nearly half of the subsequent papers on pseudoaddiction that did disclose pharmaceutical support (9 of 22) list Purdue Pharma. From the 12 papers that support and elaborate on pseudoaddiction as a true clinical entity, 4 list pharmaceutical industry support. None of the six papers that dissented or questioned the construct validity of pseudoaddiction listed pharmaceutical support.

## Proponent Versus Opponent Elaborations on Pseudoaddiction

### Articles Contributing to the Concept of Pseudoaddiction

Most of the 12 articles promoting pseudoaddiction as a true clinical phenomenon [15, 35–45] stated that aberrant drug-related behaviors (e.g., drug-seeking and other behaviors suggesting addiction in patients reporting pain) should generally be interpreted as more likely representing pseudoaddiction than true addiction, on behalf of ensuring adequate pain treatment. Positive drug screens (for non-treatment opioids or illicit substances) and other forms of addiction risk assessment are suggested to not be reliable proof of addiction, since such tools are not considered to be capable of adjusting for pseudoaddiction. This stance is adopted because the nature of the internal motivation for analgesic misuse (and not objective measurers of drug intake, or aberrant drug use patterns) is suggested to be the key factor that distinguishes pseudoaddiction from addiction. Patients reporting unrelieved pain as their reason for engaging in aberrant drug-related behaviors are understood as pseudoaddicts, and those stating they are seeking euphoria are understood as addicts. In 2003, Elander et al. formalized a means to discriminate between pseudoaddiction versus addiction, in which both syndromes are actually based on the same 12 criteria from the fourth edition of the Diagnostic and Statistical Manual (DSM-IV) for substance dependence. The presence of three or more of the DSM-IV criteria could pinpoint either pseudoaddiction or drug dependence (i.e., addiction). But, if the DSM-IV criteria occurred in association with pain or attempts to control pain, they are deemed pseudoaddiction, hence a reflection of under-treatment of pain. If they occurred in the absence of pain or involve analgesic use for purposes other than pain control (e.g., mood-altering effects and euphoria), they indicate true addiction [35].

To the extent that discerning pseudoaddiction from addiction based exclusively on patient’s subjective reporting of pain could be problematic, proponents of pseudoaddiction have espoused an additional means of confirming pseudoaddiction by addressing the under-treated pain with more opioid analgesics, and observing resolution of problem behaviors [18, 46]. Also, while pseudoaddiction is understood as being different from true addiction, it might, however, lead to true addiction, if not treated vigorously with opioid analgesics [43].

Notably, as the opioid epidemic has increased in severity and scope in recent years, attitudes about pseudoaddiction, including among authors who have espoused is existence, have evolved. For example, Passik et al. (2011) indicated that “use of the term expanded beyond the scope initially intended,” essentially being used to explain too wide a range of aberrant behaviors, too many types of patients, in too many settings. In addition, accumulating clinical experience with opioids by many pain experts had produced the realization that increasing opioid doses is often not the most effective way to reduce pain [42].

### Articles Refuting Pseudoaddiction

The major theme from the four articles concluding that pseudoaddiction is not a true clinical phenomenon [47–50] centered on the criticism that pseudoaddiction remains untested and uncharacterized as an objectively confirmable diagnosis. Instead, pseudoaddiction operates as a “clinical label without specific therapeutic, predictive, or diagnostic value” (Chabal et al., 1998) and is used to rationalize problem behaviors. These authors pinpoint the difficulty in objectively assessing motivations for aberrant drug-related behaviors, or pain, and suggest that behaviors themselves rather than self-reported motivations for the behaviors should be used as the key diagnostic information.

### Pseudoaddiction as a Social Phenomenon

Two articles espoused the perspective that pseudoaddiction (and to some extent addiction itself) is best understood as a social rather than biological construct, in which the label says more about the judgments and motivation of the diagnostician (or society) than the patient [51, 52]. The movement to frame pain management as a human rights issue rests on moral and clinical judgments about who has pain and who needs or deserves opioids. By defining pseudoaddiction (need for pain treatment) as distinct from addiction (compulsive, harmful use), there is a separation between “the ‘natural’ consequences of ‘proper’ use of opioid medications by ‘legitimate’ patients” from those with addiction [51]. The distinction between addiction and pseudoaddiction is viewed as problematic since it requires differential ethical and clinical responses [52] to “separate out ‘bad’ drug-seeking addicts from ‘good’ undertreated pain patients in the face of behaviors that are virtually indistinguishable” [51]. The claim that aberrant drug-related behaviors in pain patients are caused by the under-treatment of their condition can be extended to the behavior of “addicts,” but given that addicts are typically understood as antisocial and criminal, their behavior is viewed as a sign of their own psychology rather than the result of a particular social context [52]. In essence, pseudoaddiction is understood as the social judgment of blame on the physician for not giving opioids to patients when they should, while addiction is blame put on the patient in wanting opioids when they should not.

## Conclusion

Pseudoaddiction is a quarter-century-old concept that has not been empirically verified. Although no evidence supports its existence as a diagnosable clinical entity with objective signs and specific treatments, the term is widely accepted and proliferated in the medical literature as an “influential educational concept commonly used in pain management lectures” resulting from the “remarkable influence” of one case report [42]. Nevertheless, supporters of the concept acknowledge that differentiating pseudoaddiction from true addiction is difficult since the underlying behaviors in both (drug-seeking) are indistinguishable. The distinction then is understood as resting on the idea that pseudoaddiction patients cease aberrant drug-related behaviors and opioid misuse after their pain has been effectively treated [18, 46]. However, even this description of pseudoaddiction does not ultimately address how it can be reliably teased apart from addiction since “true addicts” will refrain from drug-seeking at least temporarily after receiving opioids, or as in the context of opioid maintenance treatment (e.g., with methadone) as indicated for opioid addiction.

The existence of pseudoaddiction, and its distinction from true addiction, is understood by proponents as being based on the patient’s reported motivation for pain relief (e.g., if their behavior results from pain, then they have pseudoaddiction, not addiction). The reliability of this conceptualization seems to hinge on the assumption that addiction and pain do not co-occur (unless one can comprehend the possibility that a patient can have fake addiction and true addiction at the same time!). However, it is not the case that pain and addiction are mutually exclusive conditions, and no clear evidence exists that having pain protects against the genesis or expression of addiction [53]. Numerous studies have found high rates of pain and opioid addiction comorbidity, while a key and nearly universal symptom of opioid withdrawal, in opioid addiction, is pain [9, 54–60]. The prevalence of addiction associated with long-term opioid treatment is difficult to assess, and estimates from various studies have varied greatly due to differences in study design, sample size, study duration, inconsistent definitions of addiction, and professional orientation of the investigators [9, 56]. A comprehensive review by Højsted and Sjøgren (2007) found that the occurrence of opioid addiction varied from 0 to 50 % in chronic non-malignant pain patients. These authors suggested that studies relying on DSM-III and IV criteria may overestimate addiction prevalence due to inclusion of tolerance and withdrawal in the criteria, which are “normal physiologic responses that often occur with the persistent use of certain medications” [56]. However, Boscarino et al. (2011) found that prevalence of lifetime opioid use disorders among patients undergoing long-term opioid therapy was virtually the same regardless of applying DSM-5 criteria (34.9 %) or DSM-IV criteria (35.5 %) [54], even though the newer DSM-5 criteria do not allow tolerance and withdrawal to count toward addiction if the patient is receiving opioids under “appropriate medical supervision.” Furthermore, a recent comprehensive review of the literature indicates that a history of opioid misuse is associated with hyperalgesia, suggesting that although short-term opioids effectively treat pain, long-term opioid use and/or addiction may actually cause or exacerbate pain [61•].

Regardless of the validity of pseudoaddiction, pain is a clinical phenomenon that remains a significant public health and scientific challenge. For severe, short-term pain, involving acute disease or injury detectable on physical exam or other objective testing, opioids have been widely accepted and empirically confirmed as effective in controlling pain and providing comfort. The following question may then be posed: Has the concept of pseudoaddiction introduced in 1989 made pain treatment any better or less challenging?

A primary difficulty in measuring pain is its highly subjective nature that is influenced by many cultural, situational, and individual neuropsychological factors [62–64]. Given the large degree to which pseudoaddiction does not distinguish itself from addiction, except based on subjective reporting of pain, and the extent to which opioid addiction is associated with or may even cause subjective pain, it is unclear how the application of pseudoaddiction has further enhanced the clinical assessment and management of pain. Clinical applications of pseudoaddiction may be particularly detrimental in the setting of chronic pain, where chronic opioid therapy has not been clearly demonstrated to improve functional status or quality of life measures [65, 66], or to be efficacious enough to warrant the serious risk of overdose or addiction [28, 30, 31, 33••, 67, 68••, 69]. Even without producing opioid addiction, pharmacologic tolerance (desensitization of analgesia) and hyperalgesia (sensitization to pain) are common in chronic opioid therapy, developing as early as 1 month after starting opioids [30, 67, 70]. But then, even if pseudoaddiction may be more appropriately and constructively applied if limited to the context of severe, acute pain involving an objectively measurable source of pain (e.g., when the risk of confusing pain with true addictions is minimal), it remains unclear how the construct has clinical utility. In other words, what is the benefit of labeling acute pain with a term other than “pain” that falsifies, or dismisses the potential presence of another brain disorder?

Characterizations of pseudoaddiction have also not addressed the fact that mental illness comorbidity is high among chronic pain patients, where psychiatric illness is a risk factor for chronic pain, analgesic abuse, and drug addiction in general [27•, 71–75]. Pain and depression frequently co-occur and, in some patients, may have the same biology [72, 74]. At the same time, depression and other mental illnesses are also risk factors for opioid dependence and other addictions [27•, 73, 76], putting those who are depressed, and more likely to seek chronic pain treatment, at especially high risk for analgesic abuse. Patients at high risk for poor outcomes on chronic opioid analgesics (i.e., those with mental health and substance use disorders) are actually more likely to receive high-dose, long-term opioid therapy—a well-replicated clinical epidemiological observation termed by Sullivan as “adverse selection” [68••, 77]. Since pseudoaddiction justifies opioid use in patients who demonstrate robust drug-seeking behavior with persistent pain complaints (e.g., in patient groups that may also have high densities of mental illness and addiction vulnerability), it is possible that clinical practices utilizing the pseudoaddiction concept may have inadvertently contributed to adverse selection.

In conclusion, we find no empirical evidence yet exists to justify a clinical “diagnosis” of pseudoaddiction. The renaming of pain with a term that essentially means “fake addiction” and serves to dismiss addiction as part of the clinical differential diagnosis is a construct that is conspicuously and uniquely attached to opioid therapies which are extremely addictive analgesics, among many other effective, evidence-based strategies for analgesia that are far less addictive. If pseudoaddiction is to remain an influential clinical construct that is taught in medical schools and textbooks, its usage and clinical acceptance need empirical support, with evidence-based disambiguation from addiction, and delineation of its treatment implications. However, to the extent that a diagnosis of pseudoaddiction relies on a self-report of pain (that is still essentially not objectively measurable) as the motivation for drug-seeking, it is not clear how rigorously it can ever be proven or disproven in human research. Even achieving objective, reliable modalities for measuring “real” pain in human beings (e.g., via neuroimaging) would not solve the issue since real pain and addiction can co-occur, and often do co-occur, in the context of opioid addiction. Instead, future studies in animals or humans that explore whether the presence of pain in general versus certain subtypes of pain (categorized by source, quality, and duration), occurring premorbid to addiction, are protective against versus predispose to future opioid addiction, may be a more fruitful approach to empirically validating pseudoaddiction. Ultimately, conclusions about the potential benefits or harms produced by the clinical acceptance and utilization of pseudoaddiction may require studies examining how clinical adherence to the construct impacts the correct identification, prevention, and treatment not only of pain but opioid addiction, and secondarily quality of life and mortality outcomes. Research should also examine the extent to which the concept may serve as a way for some providers to apply the diagnostic label of pain to legitimize opioid use in “good” patients and, in some cases, to secure reimbursement and avoid regulatory scrutiny of opioid treatment in these patients, so that they do not have to deal with “bad” opioid addicts, for whom opioid maintenance treatment is highly regulated, time-demanding, stigmatized, and uncovered by insurance [27•, 51, 52]. Whether the introduction, and subsequent proliferation and clinical application of pseudoaddiction played an unintended if direct role in inciting or propagating the current prescription opioid epidemic, or whether it reflected a medical culture that was already adversely changing to create the epidemic, remains an unanswered question. Regardless, it is hard to conclude from this review and the context of the current prescription opioid epidemic that pseudoaddiction is an objective, evidence-based diagnosis that has been clinically beneficial to patient lives. Instead, it may be most beneficial to retire the term and understand patients as simply having pain, opioid addiction, or very often both, and designing treatment strategies that best account for and balance the competing risk-benefit treatment concerns that these brain conditions imply.
